# Coproducing Health Information Materials With Young People: Reflections and Lessons Learned

**DOI:** 10.1111/hex.14115

**Published:** 2024-06-16

**Authors:** Alice Faux‐Nightingale, Glenys Somayajula, Charlotte Bradbury, Lucy Bray, Claire Burton, Carolyn A. Chew‐Graham, Aaliyah Gardner, Alex Griffin, Helen Twohig, Victoria Welsh

**Affiliations:** ^1^ School of Medicine Keele University Newcastle‐under‐Lyme UK; ^2^ Faculty of Health, Social Care and Medicine Edge Hill Ormskirk UK; ^3^ Media Studies, School of Humanities Keele University Newcastle‐under‐Lyme UK

**Keywords:** Covid‐19, Long Covid, patient and public involvement and engagement (PPIE), young people

## Abstract

**Background:**

This paper describes and critically reflects on how children and young people (CYP) acted as public advisors to coproduce health information materials about Long Covid for younger audiences. This work was underpinned by the Lundy model, a framework which provides guidance on facilitating CYP to actively contribute to matters which affect them.

**Methods:**

Coproduction activity sessions took place with CYP in schools as well as video conferences with a CYP stakeholder group and CYP with Long Covid. Activities encouraged CYP to focus on the content, format, and design of materials and used problem‐based and collaborative learning to encourage engagement with the project. Using a range of methods and open discussion, CYP codesigned a series of Long Covid health information materials for younger audiences.

**Results:**

Sixty‐six CYP (aged 10–18), and two young adults were involved. CYP codesigned specifications for the final materials and provided feedback on early designs. The project led to the development of a series of health information materials targeted at CYP: a short social media campaign with six short videos and a 12‐page illustrated leaflet about Long Covid; released on social media and distributed in local area. All the CYP were positive about the project and their involvement.

**Discussion:**

Involving CYP led to the development of innovative and engaging information materials (influence). Developing rapport was important when working with CYP and this was facilitated by using approaches and activities to establish an environment (space) where the CYP felt comfortable sharing their views (voice) and being listened to (audience) by the adults in the project. Working with external groups who are willing to share their expertise can help the meaningful involvement of voices ‘less heard’.

**Public Contribution:**

One CYP coapplicant contributed to the project design and facilitation of PPIE sessions, 64 CYP were involved in the PPIE sessions to design and feedback on materials. Two young adult media producers worked with CYP to produce these materials, another CYP supported this process. Three public contributors were involved in the preparation of this manuscript.

## 1 Introduction

While children and young people (CYP) with Covid‐19 usually experience mild illness and fewer serious complications than adults [1], some experience persistent symptoms [2] commonly referred to as Long Covid [3]. Long Covid, describes symptoms which continue 4 weeks after acute Covid‐19, including ‘ongoing symptomatic’ (or ‘postacute’) Covid‐19, symptoms between 4 and 12 weeks after initial onset, and ‘post‐Covid‐19 syndrome’, symptoms longer than 12 weeks. Symptoms are variable and can affect a wide range of systems within the body [4]. A range of symptoms have been reported in CYP with Long Covid, commonly including fatigue, breathing difficulties, headaches, myalgia/arthralgia, and sensory problems [5], and these symptoms can fluctuate in intensity and new symptoms can develop after the initial infection [6]. Long Covid can substantially affect CYP's daily activities, quality of life, development, well‐being, and participation in education and social activities [7, 8, 9].

Research about the lived experience of CYP with Long Covid highlights uncertainty and a lack of information about Long Covid and how it affects CYP. This can be hard for families when seeking support and treatment for symptoms and has an emotional impact on CYP and their parents [10]. It is important that health information is shared with CYP and families in a way that they can access, understand and use to shape decisions and choices. Working with CYP and families to develop health information either through consultation or coproduction is more likely to lead to materials which are accessible and engaging.

Patient and public involvement and engagement (PPIE) is defined in this project as working collaboratively with the public to codesign and coproduce accessible and engaging materials to inform the public about scientific research. PPIE is good practice in all parts of the research process and is encouraged by the National Institute for Health and Care Research (NIHR) in the United Kingdom (UK) [11]. Meaningfully including PPIE in projects focussed on dissemination ensures that the final outputs address relevant problems and that activities and materials best meet the needs and interests of this population [12, 13, 14, 15]. Furthermore, it can also positively affect the people who take part in the PPIE activities and, for CYP, can lead them to feel empowered, provide an opportunity to develop new skills, and increase their confidence in communicating with adults [15, 16].

PPIE with CYP should be led by their needs, interests and preferences [15] and should give the CYP agency over the work and their activity within the project. There is increased recognition within the literature that any approach to PPIE needs to be meaningful and based on the preferences of CYP and the needs of a particular project [17]. However, underpinning all PPIE with CYP are the essential principles that CYP are supported to communicate their views, are heard, and have their thoughts reflected in the project. Article 12 of the United Nations Convention on the Rights of the Child is clear that CYP have a right to express their views about issues that affect them and have those views appropriately considered and acted upon [18], and so it is important that the involvement of CYP is core to the development of resources and health information materials that are being produced for them. The Lundy model of participation [19, 20] considers this article practically and breaks it into four key elements which should be considered when working with CYP to enable them to meaningfully participate: Space—allowing CYP a place where they can safely and openly express their thoughts; Voice—giving CYP the information that they need to take part in discussion and the option to control how they take part; Audience—ensuring that CYP are communicating to people who can act on their views and comments; and Influence—making sure that CYP's views are considered and enacted where appropriate [19]. This model provides a strong foundation and a useful frame for teams to critically consider when working with CYP.

This paper describes and critically reflects, with reference to the Lundy model, on PPIE practices with CYP within the project: ‘Symptom Patterns and life with longer‐Term Covid‐19 in children and young people: co‐production of long‐Covid resources for children and young people’, the SPLaTToon project. This project was developed from the origin study: Symptom Patterns and life with Longer Term Covid‐19 in CYP [21]. Participants in this study identified a lack of information about Long Covid for young people, and these comments informed and were critical to the initial development of this project. In this paper, we pay particular attention to how activities and the setup of the project enabled CYP to contribute to the design and production of health information materials for younger age groups. We have included details according to the GRIPP short form for the reporting of PPIE activities [22], see Supporting Information Material S2.

## 2 Materials and Methods

The project took place between April–September 2023 and involved working with CYP to codesign and coproduce a series of health information materials about Long Covid. The majority of the work took place June–July with the later months set aside for printing and dissemination.

### 2.1 CYP

CYP in the earlier research project, with existing knowledge of Long Covid, were invited to take part in this project. Potential schools were selected by Keele and North Staffordshire Teacher Education, Shaw Education Trust, who facilitate teacher training placements, and the lay coapplicant, as local schools likely to engage in the project. Students carrying out work experience at Keele University School of Medicine were also invited to join in the design sessions. Following suggestions from CYP, a young person from a local acting group, selected for age and comfort acting for social media, was enlisted to appear in the short films.

The project team worked with two media producers to produce the final health information materials. These were media students recruited from the University Media department as young adults with the skills needed to produce health information materials and experience of working with social media, which was considered an important means of communication for CYP. The media producers supported sessions and contributed their own thoughts to the materials based on their experiences and expertise.

### 2.2 Coproduction Sessions

SPLaTToon was structured around two rounds of activity sessions with CYP, with time between and after the sessions for the media producers to develop the materials, and additional input from CYP throughout, full project timeline provided in Appendix S1. Sessions were held in a primary school, a secondary school, at the university, and online using video conferencing software, Microsoft Teams and Google Meet. Sessions were facilitated by at least two members of the project team (including health researchers, young lay coapplicant, and the media producers), facilitators varied between sessions.

Session activities to teach young people the tenets of research dissemination were based on social constructivist theories of learning, using the established educational theories of problem‐based and collaborative learning [23, 24]. Sessions were principally group sessions as this was felt the best format for the social constructivist pedagogical approach taken. CYP were provided with the problem of how to disseminate information about Long Covid appropriately to their age group, and collaboratively coproduced ideas for the materials to address these needs.

#### 2.2.1 In Person Sessions

Initial school sessions began with a short careers presentation about research, information about Long Covid, the SPLaTToon project, and information about what would happen in the session. Session activities were exploratory and encouraged CYP to share their knowledge of Long Covid, information relevant for CYP, and the best way to present health information to CYP. Activities were designed to foreground CYP's abilities, facilitate democracy, and provide some choice in how they shared their views [25].

**Figure 1:**
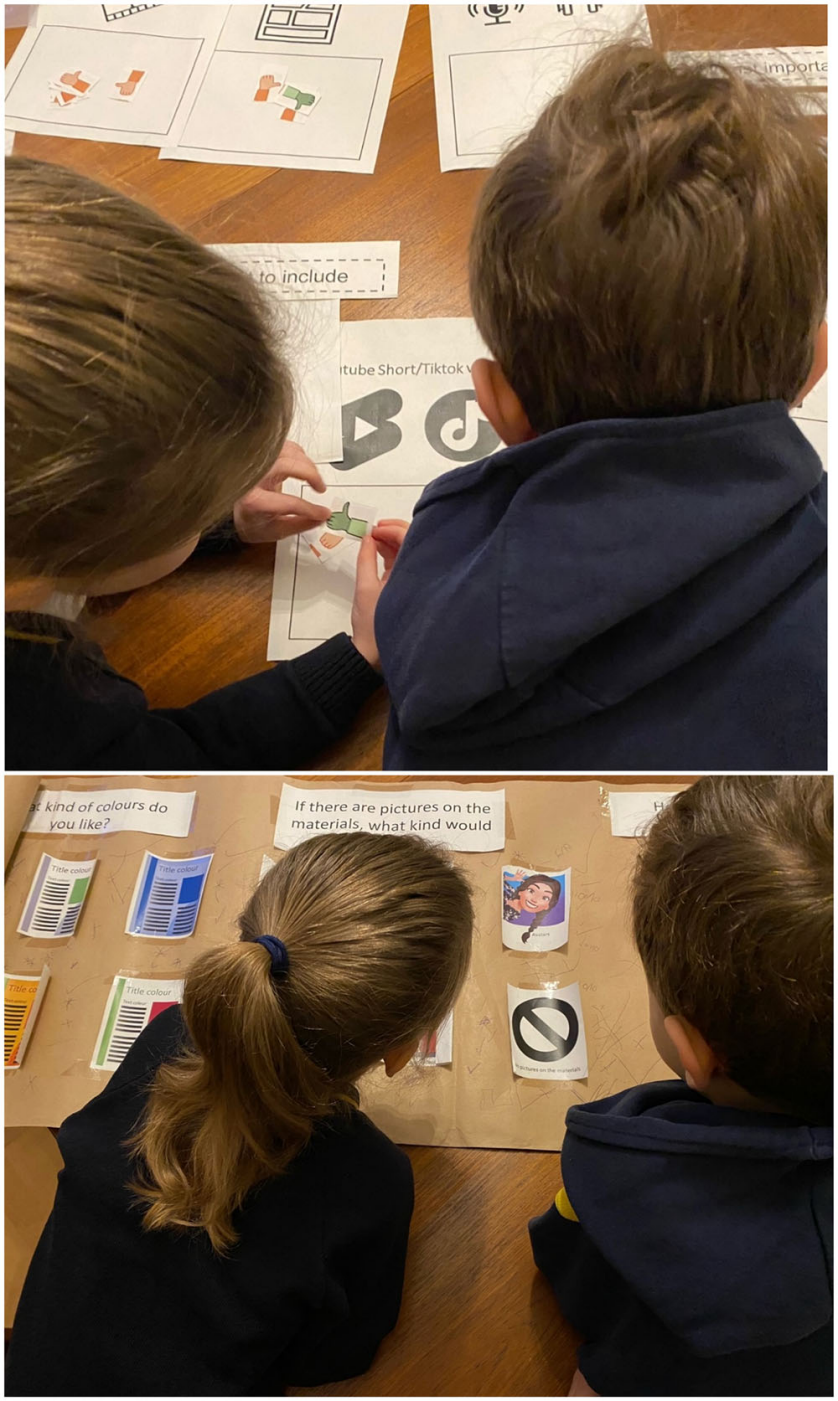
Photographs of CYP demonstrating some activities from the first PPIE session (reproduced with consent).

Sessions with younger children (aged 10–11) took place with a whole class. Sessions were broken down into a series of three activities undertaken in smaller groups to facilitate social interaction and problem solving to produce the materials: content, presentation, and design, see Supporting Information Materials S3. Activities and materials were designed specifically for this session in connection with a primary school teacher and playleader. Activities emphasised learning through social interactions [25] by providing experiences for visual, auditory, and tactile learning styles. Activities involved physically engaging with materials, see Figure 1, with minimal writing to reduce strain on CYP uncomfortable using this format of communication. Activities were facilitated to allow CYP to engage verbally if they did not want to engage practically. Blank paper was available if CYP wanted to contribute additional ideas to the project, researchers offered to write notes as necessary.

A focus group style approach was taken with older (secondary school) CYP as the group sizes were smaller which made group discussion easier, and it was thought that older CYP may be more willing to discuss their thoughts with the project team. In these sessions, CYP were provided with information about how Long Covid affects CYP and introduced to the ‘problem’ of there being few health information materials available to young people. Facilitators encouraged the group to discuss the problem and ways that they would engage with health information, to develop specifications for future materials.

Alternative activities were provided for CYP who were unable to leave the classroom if they did not want to participate, allowing them to choose not to take part.

#### 2.2.2 Virtual Sessions

Activities were redesigned for the virtual sessions where CYP would not have access to materials.

Information about the project was shared using the same short presentation as used in the primary school, then CYP were asked the same questions in a focus group style session. CYP were encouraged to take ownership of their participation and engage in any format, including by typing responses, based on their preferences.

All CYP were offered the opportunity to contact the research team separately, enabling them to continue to offer ideas if they had felt restricted in the session.

#### 2.2.3 Material Coproduction

Following these sessions, media producers produced initial drafts of the materials based on the specifications outlined by CYP. Media producers identified areas which required further input and development from CYP, and these early drafts and questions were developed into activities for a feedback session, see Supporting Information Material S3, to allow CYP to ‘quality check’ the materials, and give feedback and suggestions for change.

At the end of the feedback session, a short evaluation sheet, not designed with CYP due to time restraints, was given out to capture CYP's reflections about the sessions, supporting the CYP in reflecting on their activities, and providing information which will be used to develop future projects. We considered that young CYP would likely need support with their reflections, and this informed our evaluation form. Three questions were presented as Likert scales with accompanying ‘emojis’ faces, these asked: What the CYP thought about the sessions; whether they would like to see the final materials; and whether they would take part in similar activities again. Two free text answers asked CYP to write something about the project that they had enjoyed and if there was anything that the research team could do to improve future projects and engagement activities. All CYP were told that they could answer freely and complete the sheet independently, although facilitators were in place to support if requested. To facilitate honest responses and allow CYP to be comfortable to express their thoughts, all sheets were anonymous and CYP delivered them to a pile at the front of the classroom where they were not looked at by the research team until after the session. Online participants were emailed these evaluation sheets to ask for their feedback, which they could return via email.

CYP who took part in the coproduction activities in this project were provided with project participation certificates and stickers created by the research team as a thank‐you for their contributions. The media producers and CYP who were directly involved in the production of the materials were paid the NIHR INVOLVE rate (£25/h) for their contributions [26].

### 2.3 Ethics

As SPLaTToon was a PPIE project, carried out “‘with’ or ‘by’ members of the public rather than ‘to’, ‘about’ or ‘for’ them” [26], no ethical review was required for the activities. This was confirmed via submission to the Faculty Ethics board at Keele University. However, the project team took an ethical approach to the project, and within the sessions, CYP were supported in their understanding of the project and in their capacity to agree or decline joining in the activities.

As part of this project, contributions from CYP were anonymised and not attributed to any of the individual participating groups. While CYP's thoughts and feedback were used throughout the design and production process, no data was shared between groups other than through occasional discussion of concepts and ideas which had been raised in the activities to prompt further discussion.

Not having ethical approval meant that we did not receive consent from CYP to publish any of their responses to the activities. As a result, this paper is based on researchers' observations. Some of the CYP who worked on this project cowrote this paper, they have consented for their words to be used and are listed as authors to reflect their contributions.

### 2.4 Results and Outputs of the SPLaTToon Project

In total, 66 CYP with and without Long Covid, aged between 10 and 18, worked on the project, see Table 1.

**Table 1:** Summary of CYP involved with SPLaTToon.

CYP	Number of CYP	Age range (years)	Gender	Ethnicity
Lay coapplicant, involved in developing the project and facilitating sessions	1	17	M	White: British
Young people with Long Covid (and their parents), participants in the SPLaT‐19 study	2 (+2)	10, 14	2F (2F)	White: British and Mixed: White & Black Caribbean
Primary school students, in teams named: ‘Mclilfecre’, ‘Monn Ldcj’, ‘Gamer Greg’, ‘Big banana’, ‘Smurfs’, and ‘Blood red’ (State school; 6th decile of deprivation^a^)	47	10–11	Mixed (unrecorded)	Mixed (unrecorded)
Secondary school students (Selective, grammar school; 5th decile of deprivation)	4	11–13	3F, 1M	Unrecorded
Work experience students	4	16–18	3M, 1F	White: British
Members of the NIHR CRN WM Young Research Champion's Group, involved in the SPLaT‐19 study	7	11–17	Mixed (unrecorded)	Mixed (unrecorded)
Young amateur actor, involved with video filming and production	1	13	F	White: British

^a^ Deprivation data as reported for the school postcode by the Ministry of Housing, Communities & Local Government [27].

CYP considered a wide range of elements important for communicating with younger audiences and these were used to develop a series of specifications used to guide development of the final materials, see Table 2. Within the sessions, CYP also discussed ways to increase reach to young people. They suggested that TV would be a good way to disseminate to CYP, particularly through youth programs like Newsround or Operation Ouch. CYP also described factors that would make social media videos more appealing to young people, for example: including ‘easter eggs’ in videos; having an attractive video thumbnail; and increased search optimisation to ensure that videos appear high in search results. The CYP had many insightful ways to increase the reach of the final materials, however, many of these ideas fell outside of the budget or time constraints of the project and so we were not able to include them in the final materials. Where difficulties with budget or time constraints were identified during discussion, session facilitators used this as a talking point with the group and explained how limited funds may restrict enacting the suggestions. CYP were encouraged to think about ways to work around the restraints while still maintaining interest and access for CYP.

**Table 2:** Specification comments from CYP and how they were integrated into the final information materials.

CYP comment	Reflection in final materials
Include information about Long Covid and how it affects CYP, but make sure that the information is not so much that it provokes worry or stress.	Materials included background information about Long Covid and topics that CYP considered important. Information focused on common symptoms and experiences, and where CYP could go for help to reduce the risk of worry or stress for CYP.
Share information through social media platforms commonly used by CYP, e.g., TikTok and YouTube. Interest for CYP could be enhanced by: including ways for CYP to interact with the video; structuring the video to incorporate a surprise or an answer to an initial question, which would ensure that viewers watch to the end; and use of paid adverts to increase the number of people who see the video.	Materials produced for social media were disseminated on TikTok and YouTube. Videos were introduced around questions, as suggested by CYP. Videos were not shared using paid adverts as this was not factored into the project budget.
Information should be provided in short snippets, not in long form.	Information was broken up into key topics. Topics were presented in separate videos on social media and were in separate sections on the leaflet. Social media videos were all under a minute in length, except for one which was 1:13.
Offline materials should be available for CYP who are unable to access the internet. Information that can be read with parents could support CYP in accessing information.	An illustrated leaflet was produced and has been distributed in local schools and GP practices. This can be read by parents and CYP as necessary and promotes access for CYP without access to the internet, including cases of digital deprivation.
Materials should be colourful, but not so colourful that it becomes hard to read or distracting.	CYP suggested that a monochromatic colour scheme was an appropriate level of colour for the materials. They selected and refined a colour scheme which was applied consistently across all materials. CYP were also asked about colours of text and illustrations and commented on appropriate levels of colour throughout (e.g., text and background colour, colour of illustrations).
Cartoon characters are a good way to visually convey information.	CYP provided feedback on initial character design for the materials, making suggestions about design and representation, e.g., realistic skin tones/hair colours were better for these materials than bright, unrealistic colours.
Materials should be clear where the information comes from to increase credibility. Researchers, doctors, and CYP with Long Covid are trusted sources. Logos are a good way to identify associated, trusted organisations.	Social media videos had information presented by a CYP, doctor and a researcher and included quotes from CYP with Long Covid and their parents. All materials included references to the university and included a link to the university project website for further information to promote credibility.
Materials should be as accessible to as many people as possible, particularly considering groups with visual impairments, audio impairments, and dyslexia.	Materials were produced in two formats to increase accessibility as much as possible. In the social media videos, information was presented verbally, and subtitles of the script were also included on the screen. The leaflet was produced as a printed document, but a pdf was provided on the website to support the use of screen readers. The colour scheme and font for the materials were selected by CYP with dyslexia to be appropriate for their needs, but the media producers also considered dyslexia style guides [22] to ensure that the final materials were as accessible as possible.
Different materials should be produced for younger and older CYP who will have different needs and interests.	Due to funding limitations, we could not meet this criterion in addition to the others. Older CYP had suggested that they would be more likely to look for information on websites like NHS website, etc., so would be less likely to engage with materials like this. With this in mind, we focused the project on the early‐mid CYP ages.

The media producers worked with these specifications and feedback to produce a series of health information materials to tell CYP about Long Covid. The final materials were:

- A social media campaign for CYP consisting of six short videos and still image posts for social media platforms like TikTok and YouTube.
- A 12‐page illustrated leaflet for CYP and their parents to be shared in schools and other public places.

These materials are displayed on project website: www.keele.ac.uk/splattoon, and are available to the public on TikTok and YouTube where they will circulate and continue to be accessible to the public. Leaflets have been printed and circulated within the local area, but the pdf will remain on the website for download and further printing as needed.

The groups spoke thoughtfully about accessibility, diversity, and the importance of information being credible. CYP were particularly passionate about ensuring that resources were accessible and talked about how ways to make the materials appropriate for a range of audiences. Those with dyslexia in the group spent a long time talking about design and proposed font/colour combinations with a view to making sure that they could also engage with the materials, and their classmates were keen to support them. When the CYP‐approved designs were checked against accessibility checklists and guidance, they met the criteria well [13, 28], including level AAA for the NHS colour contrast standards [29, 30] (the highest scoring for accessibility), and this reflected the level of care and attention that the CYP put towards considering this design element. Despite social media being the preferred media output, CYP also thoughtfully considered digital deprivation and were thus keen that an offline leaflet was also produced.

### 2.5 Evaluation and Reflections on the Project

CYP were asked to provide feedback about taking part in the project. Their responses were overwhelmingly positive with most CYP saying that they enjoyed taking part in the sessions and no‐one responding negatively. Nearly all said that they would take part in similar consultation activities again.

When asked to write what they enjoyed about taking part, the most common responses were that the sessions had been fun, and they enjoyed the activities. However, CYP also appreciated that their ideas were being included in a project and that they were helping to design materials which were going to be used to share health information with their peers.

Working with the Media Department and Internships team at Keele University provided a way to include students who had the skills to work as media producers and had equipment and technical support available to take on the project and complete the final materials to a high standard. The media producers talked about the personal benefits of their time on SPLaTToon in terms of ‘technical [and] workplace skills’, but further discussed how they had come to realise the importance of working with CYP and clinicians together when producing health information materials for young people. Data extracts with author's initials are given to illustrate our discussion^1^.

I found working with doctors, researchers, and children with Long Covid inspiring and the project highlighted the importance of sharing these health materials with wider audiences and how necessary accommodating targeted audiences is when health information is involved.

(AGG)

For them, being able to be part of sessions where they could talk to and work with CYP was a key part of the project, as was producing materials in a format that CYP were likely to engage with.

I feel, similarly, that sharing information like this over TikTok and similar platforms is essential for projects like this. When trying to engage with young people, you have to go where they are.

(AG)

The young actor found that this was a positive learning experience for them on many levels. They enjoyed the opportunity to go to a university, ‘I hadn't been on a university campus before and did not know what to expect. I enjoyed walking around and seeing the facilities that were there’. (CBr), The opportunity afforded them with their first‐time filming for a national audience and an increased awareness and interest in the work that goes on behind the camera.

## 3 Discussion and Lessons Learned

To produce health materials which are accessible and engaging for CYP, it is important to listen and include them in the design and production process [31]. The SPLaTToon project team engaged CYP of a range of ages and experiences and coproduced a series of Long Covid information materials for CYP. This discussion will reflect on our work with CYP according to the key principles of the Lundy model of participation, which have been effectively used to consider CYP's participation in a variety of settings [32, 33, 34], and the way that activities and an environment can be used to promote a demographic space for CYP which supports their participation and collaboration to problem‐solve and develop materials [23, 24, 35]. We use the elements from the model: Space, Voice, Audience, and Influence [19] to consider how CYP were able to meaningfully contribute and have their views heard and enacted in the development of these health information materials.

### 3.1 Facilitating ‘Space’

Space within the Lundy Model is described as establishing a setting where CYP can safely and openly express their thoughts [19]. Similar projects have considered appropriate locations for research with CYP, including schools [36] and youth centres [37] as these can be familiar environments for CYP. Working with CYP in school spaces allowed us to include CYP who were unfamiliar with PPIE or research, ensured that CYP were not required to travel or pay to take part in the session, and meant that they were in spaces that they already felt familiar and comfortable. School rooms were big and well equipped for the session and CYP were in an established group that they felt comfortable talking and working with, although within the constraints of an environment where CYP are historically expected to be compliant and there are typically right and wrong answers. While working with a youth centre may have mitigated against established boundaries and relationship dynamics between school staff and children [37] working in a school environment increased access to participation as there was no requirement for parents to attend or provide their children with additional transportation [38].

To facilitate open discussion, we established safe and open spaces for CYP to engage with the project. We made it clear at the start of the session that there were no wrong answers and CYP's contributions were welcomed and valued within the project. Facilitators maintained an open discussion with CYP and made minor adjustments to each activity to best meet the needs of the groups they were working with. The space made it easy for us to carry out our activities and these were successful and kept the class engaged and excited for the whole time. The series of activities also worked well on a class level, as it broke the full class into manageable groups which could be more easily facilitated by the researchers.

Working in the final half term of the school year meant that we were working after all exams (SATS, GCSEs, A‐levels, and University exams) and students were not restricted by revision or exam stresses. Schools were enthusiastic about participation at this time which made organising sessions straightforward, and we had sufficient time to run the activities. However, working within the final half term meant that our second session was due to take place in the final 2 weeks of the year which coincided with schools' end of year activities so we could not organise a return visit to the secondary school. This meant that we missed out on feedback from the older age group. To avoid similar issues in the future, we advise either trying to organise the sessions to be closer together or conduct a concentrated day where CYP could also be involved with all steps of design and production.

CYP who had been involved with the SPLaT‐19 research project, participants and the CYP steering group, took part in virtual PPIE sessions instead of the in‐person sessions which were carried out in schools. Offering virtual sessions increased access to the project and facilitated their involvement in the project, as many of these CYP were spread out geographically. Virtual sessions also enabled CYP with Long Covid to control their participation levels to match their energy levels at the time, another benefit, although we acknowledge that offering virtual sessions can act as a barrier to access for people with limited access to technology. However, while virtual spaces supported CYP's access to the project, there were difficulties engaging CYP in virtual sessions in this way. In one session, CYP chose to keep their cameras and microphones off, only engaging with the questions and activities through the chat function. While they still participated and gave useful responses, involvement was limited due to lack of visual cues, and the challenge of developing in‐depth discussions over the chat function. The use of email and Teams to share documents also meant that CYP could not submit their evaluation forms anonymously, which may have influenced their responses. Future projects will need to develop activities specifically for virtual space to meet these different needs, for example developing activities to encourage CYP to talk or otherwise respond individually to questions before opening up topics for group discussion, potentially using software such as online forms or digital whiteboards that enable anonymous contributions and offer additional ways for CYP to contribute their ideas.

### 3.2 Facilitating Voice

Voice is described as facilitating children's views [19]. We know that CYP are able to identify and articulate their own health information needs [39, 40] and found that in our sessions, CYP were enthusiastic and capable of talking about the level of information that they wanted to know and the best way to present it. CYP were given information about the project, Long Covid, and the research findings to include in the materials so that CYP were able to understand their role and could meaningfully take part in the discussion. For this coproduction project, it was important for us to work with CYP and enable their participation by developing activities that were engaging and interesting [38] around the key areas necessary for the design of the materials (content, presentation, and design), and which supported an open discussion forum for CYP.

Many studies describe the benefit of using participatory activities, for example, drawing, use of sticky notes, and categorising of pictures using letter boxes, when working with CYP to identify their information needs [40, 41, 42]. The use of multiple types of methods, particularly including visual and linguistic methods, is suggested to be particularly beneficial for CYP as it allows them to engage using a range of methods of expression [43]. The primary school activities were successful at getting CYP to engage in the topic and consider the different elements needed to produce successful health information materials. Although the activities were designed to be engaging for younger age groups, it was important to remember that children may not want to participate in the prescribed activity or may prefer to participate in alternative ways [44]. Introducing flexibility into the design of the activities and facilitation on the day allowed CYP to choose the degree to which they engaged with the topic, if at all: some did the activity but no more, others used them as a springboard for new ideas and creative thoughts—having paper and pen on hand ensured that all ideas were captured. Hands on activities seemed to work best, particularly those with little reading or writing, as CYP could contribute quickly and then talk about their thoughts, as did the use of stickers and sticky notes for feedback. Although these participatory activities were not carried out with secondary school CYP or in the online sessions, this is something we would consider adapting for future projects.

It is important to ensure that CYP can choose whether they join in activities or not. Where CYP did not turn up to sessions, we did not introduce any penalties or follow them up, we continued as we had planned with those who arrived. CYP with Long Covid who had expressed interest but did not attend were offered the opportunity to contribute via email at a convenient time for them, but no‐one responded using this route. In the classroom, we recognised that attendance was more structured, but provided alternative activities for CYP who did not want to join in. These went unused, but one class teacher appreciated that they allowed CYP the choice to not take part.

While logistically the end of the academic year was a good point to work with students, running the project at this time also led to some difficulties. Finishing the project during the summer holidays meant that the materials were only available for distribution in the following academic year. Year 6 students who had been involved in the project had left the school before we were able to return the finished materials. We also found that schools were less engaged with the project after the summer holidays, and it has been hard to find out which schools the Year 6 students have moved to, to pass on the materials to. In future projects, we will be more aware of how the influences of adult timetables on project plans can limit CYP's ability to engage with projects and project outputs and develop more inclusive practices. It is advisable to expand the number of schools involved in similar projects and consider including community groups to the project and expand to include community groups, to include a more diverse range of CYP in the coproduction process. This would allow us to better identify interests and access needs for this age group.

### 3.3 Facilitating ‘Audience’

Audience, defined in the Lundy model as allowing CYP to know that they are communicating to people who can act on their comments [19], was addressed in the introductory project brief where the facilitators from the research team provided background information about themselves, the research study, and the SPLaTToon project. As part of this introduction, we were clear about what we would be doing in the sessions, why, where the comments and responses from the sessions would go, boundaries of the project (financial boundaries which would affect the outputs, that is, we could not produce a national TV campaign, and time restraints for the project) and what would happen with the final materials. This introduction emphasised that the CYP were helping to advise the research team and that their thoughts and opinions were being valued by researchers and doctors as they were experts on what was interesting to CYP. Having the feedback session helped the CYP see that their ideas had been taken seriously by the project team.

### 3.4 Facilitating ‘Influence’

It is important to establish open relationships between CYP and adults where it is clear that there is a willingness to learn and respond to CYP's thoughts, priorities, and contributions [45]. In this project, CYP were supported in having some control over the development of the health information materials produced in this project and that, as indicated in the Lundy model, their contributions would be valued, enacted where appropriate, and integral to the project [19]. CYP were given control over the content, presentation, and design of the materials, within the project constraints, and young people were also involved in the production of these materials. While it was impossible to integrate everyone's ideas and comments into the final materials, CYP's ideas and designs were summarised into a list of specifications for the media producers, and CYP were given the opportunity to provide further feedback and shape the early drafts of the materials. As researchers, we were impressed by the ideas and maturity of the responses from CYP, particularly a clear interest in inclusivity and diversity at a young age. While some ideas were predictable such as developing information for social media, some preferences such as font types were different to what we had expected the CYP to choose. This has reinforced the importance of including CYP in research processes rather than assuming that we are able to predict ideas and preferences of CYP. The only specification that we were not able to meet, due to budget constraints, was different materials for older and younger age groups, who may have different visual interests, and this reflects a limitation of the influence of CYP within this project. Furthermore, while some CYP were involved with the production of the final materials, we were not able to enable all to be involved with the production phase, due to limited time and budget. Future projects will aim to expand the budget for PPIE activities to further facilitate CYP's engagement and influence over the project. Despite these limitations, we hope that the final materials will still be of interest to a wide age range but recognise that some CYP, particularly older and much younger CYP, may find these materials more challenging to engage with.

While we worked to support access to the materials, there are areas that we were unable to address within the scope of the project. Limited funds and time made it difficult to address cultural, linguistic, or socioeconomic factors which could affect access to the resources. However, had we been able to increase the size of the project, we could have included more schools (including a broader range of cultural and socioeconomic backgrounds) and considered how we could address these barriers to support increased access to the materials, for example, providing translations of materials. A further limitation is that we did not record gender and ethnicity in the larger groups that we worked with, although we did work with diverse groups in schools and the stakeholder group. In future projects, we will ensure that these details are recorded.

### 3.5 Impact and Outcome Measurement

The project expanded the PPIE groups associated with this study to include new partners within the local community. For most of the children and young people involved in the study, particularly those in primary and secondary schools, this was the first time that they had heard about research at universities and the first time that they had participated in research projects. Given their positive responses to the project, interest in the outputs, and comments about how they enjoyed being able to contribute, we hope that this project will encourage them to engage with research in the future and perhaps participate in future studies.

While not explored in this paper, impact of this project will be assessed in the coming months using engagement figures from the online resources, for example, viewing numbers, ‘likes’ and so on, and through responses from places which have distributed the leaflets. Further work could also explore the effectiveness of these materials in communicating information to young audiences. CYP suggested that engagement could be enhanced by use of paid advertising on social media platforms, however, funding limitations to the study prevented our use of these. This could be a valuable opportunity for future projects.

## 4 Conclusions

CYP worked with the research team to coproduce a series of multimedia health information materials about Long Covid which are engaging and accessible for young people. Producing these materials with CYP provided insight into the needs and interests of this age group, which can shape research and dissemination in the future. Schools facilitated access to large groups of CYP and were keen to participate, although it was difficult to keep in touch once the pupils had moved on. Activities encouraged CYP to actively engage with coproduction and many materials from these activities have been retained for use in future projects. Focus group style discussion was harder to manage with CYP, particularly using online methods where CYP elect to keep their microphone/camera off, future PPIE work would benefit from findings ways to adapt activities to an online setting to encourage engagement.

Sessions were an insight into the base knowledge that the public have about research and health information. We went into schools expecting that children would have some knowledge about universities and Long Covid. However, we ended up spending substantial amounts of time in sessions talking about Long Covid and sharing basic information about how it affects people. This was a useful lesson in not only assuming knowledge, but also draws awareness for the need for public health information materials for CYP, to support the development of their health literacy.

## Author Contributions

**Alice Faux‐Nightingale:** conceptualization, funding acquisition, writing–original draft, writing–review and editing, methodology. **Glenys Somayajula:** conceptualization, funding acquisition, writing–original draft, methodology, writing–review and editing. **Charlotte Bradbury:** writing–original draft. **Lucy Bray:** conceptualization, funding acquisition, writing–original draft, methodology, writing–review and editing. **Claire Burton:** conceptualization, funding acquisition, writing–original draft, methodology, writing–review and editing. **Carolyn A. Chew‐Graham:** writing–original draft, writing–review and editing. **Aaliyah Gardner:** writing–original draft. **Alex Griffin**: writing–original draft. **Helen Twohig:** conceptualization, writing–original draft, writing–review and editing, funding acquisition, methodology. **Victoria Welsh:** conceptualization, writing–original draft, funding acquisition, writing–review and editing, methodology.

## Ethics Statement

As SPLaTToon was a PPIE project, carried out “‘with’ or ‘by’ members of the public rather than ‘to,’ ‘about’ or ‘for’ them”, no ethical review was required for the activities, confirmed with a faculty ethics panel. However, the project team took an ethical approach to the project, and within the sessions, CYP were supported in their understanding of the project and in their capacity to agree or decline joining in the activities. A consequence of not having ethical approval for the work is that we did not receive consent from CYP to publish any of their responses to the activities. As a result, the results of this paper are based on researchers' observations. Some of the CYP who worked on this project chose to cowrite this paper. They have consented for their words to be used and are listed as authors to reflect their contributions.

## Conflicts of Interest

CC‐G is partly funded by WM ARC and has received other grants to investigate Long Covid. The other authors have no conflicts of interest to declare.

## Supporting information

Supporting information.

Supporting information.

Supporting information.

## Data Availability

Children and young people who took part in this project did not give consent for their data to be shared publicly, so supporting data is not available.

## Footnotes

1 Quotes here have been written by young people who were involved with the project. They have consented to us using their words here and have joined us as authors on the paper to reflect their contributions.
