# Supplementary material for: Coproducing Health Information Materials With Young People: Reflections and Lessons Learned
Source: Health Expect. 2024 Jun 16;27(3):e14115. doi: 10.1111/hex.14115 (PMC11180295; doi:10.1111/hex.14115)
Supplement: Supplementary file 2 — Supporting information. [file HEX-27-e14115-s003.docx]

Supplementary Material 1: GRIPP short form

|  |  |  |
| --- | --- | --- |
| Section and topic | Item | Reported on page No |
| 1: Aim | Report the aim of PPI in the study | 3 |
| 2: Methods | Provide a clear description of the methods used for PPI in the study | 3-5 (and Supplementary Material 2) |
| 3: Study results | Outcomes—Report the results of PPI in the study, including both positive and negatives | 5-8 |
| 4: Discussion and conclusions | Outcomes—Comment on the extent to which PPI influenced the study overall. Describe positive and negative effects | 8-11 |
| 5: Reflections/critical perspective | Comment critically on the study, reflecting on the things that went well and those that did not, so others can learn from this experience | 8-12 |

Reference: Staniszewska S, Brett J, Simera I, Seers K, Mockford C, Goodlad S, Altman DG, Moher D, Barber R, Denegri S, Entwistle A. GRIPP2 reporting checklists: tools to improve reporting of patient and public involvement in research. bmj. 2017 Aug 2;358.
