# Supplementary material for: Coproducing Health Information Materials With Young People: Reflections and Lessons Learned
Source: Health Expect. 2024 Jun 16;27(3):e14115. doi: 10.1111/hex.14115 (PMC11180295; doi:10.1111/hex.14115)
Supplement: Supplementary file 3 — Supporting information. [file HEX-27-e14115-s001.docx]

**Supplementary Materials 2: Details about session activities**

**Design session**

All activities were facilitated by members of the project team who encouraged the CYP to engage openly in discussion around the topics and had paper to record any comments or thoughts from CYP, either about the topic or which addressed another topic for the project.

**Content**

**What topics should be included in Long Covid materials for CYP?**

CYP were asked about the information which they thought would be important to include in materials about Long Covid. Subject topics based on the findings from the SPLaT-19 were printed on to strips of paper and CYP were invited to rank these topics from most important to least important so that the most important/interesting topics were at the top and least important/interesting were at the bottom. Any topics that were not relevant could be removed. In discussion, CYP were asked what information they would look for, to explore any additional topics that needed to be included.

**Who do you think is best to share information about Long Covid?**

Facilitators asked CYP to think about the way that information is presented to the audience, and who they would trust information from. A selection of pictures with different people and roles were displayed on the table and CYP were given sticky stars to put next to the picture of the person that they would trust information from. Options included: Other children, A young person who has long-Covid, Teacher, Doctor, Researcher, No-one (information displayed without a presenter), Other person (CYP suggestions included a parent). Discussion explored credibility of information and what kind of information they would trust online, facilitators also asked the group how the project could make sure that the information appeared credible to young people.

**Presentation**

**What is the best way to share information with CYP?**

In this activity, CYP were encouraged to talk about how they accessed information outside of school and think about the best place to display information about Long Covid for CYP to access it. In this activity, a series of paper sheets with a range of ways of providing information were laid out on the table for the children, options included: Short film, Tiktok/Youtube shorts, Leaflet, Postcard, Poster, Website, Podcast, Comic, Art. CYP were given cards with a ‘thumbs up’ on one side and a ‘thumbs down’ on the other and they were asked to respond to each option to say whether they thought it was a good or bad way to share information with CYP. CYP were encouraged to expand on their answers with the facilitator to clarify their responses, but an ‘Other’ sheet was also provided to let CYP add additional ideas.

**Where is the best place for us to put information about long-Covid so that CYP will see it?**

In addition to thinking about the format of the information materials, we also asked CYP about places that we could display the information and where they thought we could put the information for it to be most accessible for CYP. For this activity, we gave each CYP a pack of small cards with an image and title of a series of places: School, Library, Doctors'/GP practice, Social media, Community locations e.g. scout's hut/church/youth group, and the Internet. Blank cards were on the table to allow CYP to add any other ideas. Three boxes with gold, silver, and bronze medal pictures on the side were on the table and CYP were asked to select their top three places out of their cards and put them in the ranked boxes – rankings were not fixed and if CYP wanted a joint ranking or to put more than one idea in the box, they were able to do so. A bin was provided at the side of the table, and any bad ideas could be thrown away.

**Design**

**Style conveyer belt**

A ‘conveyer belt’ was developed for the session which encouraged CYP to consider different elements which contribute to material design and to consider the importance of design when sharing information with people. This consisted of a long strip of paper broken into sections. Each section addressed a different design factor with ~8 options for the CYP to consider: Colour/colour scheme, Font, Font size, Images/graphics, and Layout. CYP were given pens and asked to score each option and talk about why they picked the options they did, although most children instead chose to tick/cross each option to say whether it was acceptable to them or not. In some cases, CYP also added notes to the sheet to add further detail to their responses.

**Ballot box for the logo**

Two logos were available for use for the SPLaTToon project. One which was developed for the SPLaT-19 project by a CYP co-applicant, and a second which was produced for the SPLaTToon grant application by a member of the project team to complement the SPLaT-19 logo. CYP were given the opportunity to choose between the logos to decide which one would be included on the final materials. They were given a voting slip with both options on the paper and were asked to put it in the ballot box. After all of the design sessions, the votes were counted and decided the final logo for the materials.

**Feedback session**

Media producers were asked to produce early drafts and designs for their materials, based on the thoughts and ideas from the initial design sessions. As part of this, they also wrote a series of questions that they wanted to receive feedback on, to help them develop the drafts further. These early drafts and questions were developed into a series of activities for the groups:

Visual designs were printed in colour and given out to the groups with post-it notes and shiny star stickers. Young people were encouraged to look at the designs and talk about what they liked/did not like about them, we asked CYP to add stars to elements that they thought were good and use the sticky notes to add comments about things that they did not like or add thoughts about what could be improved/added.

A short questionnaire about social media use was developed by the media producer to support the production of short videos for TikTok. Children were asked to talk about their social media use, any accounts which they followed, and any key features/producers they liked, to try and determine any features which would be useful for the final materials. While CYP were able to write responses on the sheet, these questions were mostly used as prompts for the facilitators to talk to CYP about these areas.

A short evaluation sheet was given out to document young people’s thoughts of the sessions. This used Likert scales with emoji descriptors to ask whether the young people had enjoyed taking part in the session, whether they were interested to see the final materials, and whether they would take part in similar activities again. If they had time and were willing, young people were also asked to write one good thing that they had enjoyed about the sessions and say one thing that we could do to make the sessions better.
